# Destination authenticity as a halo? Enhancing emotional solidarity with residents in rural tourism

**DOI:** 10.1371/journal.pone.0331644

**Published:** 2025-09-05

**Authors:** Jing Zhou, Yaobin Wang, Yaohua Wang, Haoyang Miao

**Affiliations:** School of Tourism, Northwest Normal University, Lanzhou, China; Guilin University of Technology, CHINA

## Abstract

Emotional interaction between tourists and hosts is a vital element of rural tourism, and tourists often seek nostalgia and authenticity in rural settings. Based on the cognition-affect-behavior (CAB) framework and the halo effect theory, this study constructs a hypothetical model to explore the antecedents and consequences of emotional solidarity between rural tourists and residents. Online responses were collected from 324 Chinese rural tourists. The data were analyzed using PLS-SEM. The results indicate: (1) Perceived authenticity of rural tourists has a positive impact on nostalgic emotions; (2) Both perceived authenticity and nostalgic emotions promote emotional solidarity between rural tourists and residents; (3) Emotional solidarity is a significant predictor of revisit intention. This study enhances understanding of emotional solidarity in a rural context and provides practical insights for stakeholders to foster host-guest relationships.

## 1. Introduction

The host-tourist relationship is a fundamental component of the tourism experience [1]. Positive social interaction with local residents in tourism, or the perception of such possibilities, is a key attraction for tourists [2]. Given the social nature of human relationships in tourism, the host-tourist relationship has been acknowledged as pivotal in the tourism and hospitality sectors, garnering enduring interest and examination [3–5]. The exploration of the host-tourist relationship holds particular significance in rural tourism, as rural tourism is a form of tourism characterized by high levels of emotion and interaction. [6]. This becomes especially pertinent in China, with its substantial rural tourism market, where the evolution of rural tourism has integrated profound emotional and cultural attributes influenced by traditional agricultural practices [6]. The traditional ethos of agriculture serves as motivation for Chinese tourists to engage in rural tourism, where the strong interpersonal ties are a notable characteristic of traditional Chinese agricultural society [6]. Against this backdrop, recent research has increasingly examined the dynamics of interpersonal relationships in rural tourism. For instance, Wang and Yotsumoto (2019) investigated conflicts among stakeholders in China’s rural tourism sector [7]. Wu et al. (2022) examined the daily interactions and social relationships of rural residents from a sociological perspective [8]. Furthermore, Chen et al. (2024) analyzed the phenomenon of coexistence between rural tourism practitioners and local residents [9]. However, there has been a paucity of studies examining the emotional connection between rural tourists and residents. Given the distinctive role of the tourist-host relationship in rural tourism in the Chinese context, this study seeks to quantify this relationship and investigate its formation mechanisms and positive outcomes.

The utilization of emotional solidarity in tourism has the potential to enrich the existing literature. After its inception in tourism research by Woosnam and Norman (2010), emotional solidarity has been employed to elucidate the connection between hosts and tourists [10,11]. From the viewpoint of tourists, emotional solidarity is conceptualized as the emotional linkage and rapport between tourists and hosts [12,13]. Prior research has investigated the pivotal role of emotional solidarity in various contexts, including P2P accommodation [14], religious tourism [15,16], and community tourism [17]. Nevertheless, the processes underlying the formation and consequences of emotional solidarity within rural tourism settings have been largely overlooked. Additionally, as emotional solidarity was originally conceptualized as arising from shared beliefs, behaviors, and interactions [18], numerous tourism studies have stressed the significance of interpersonal interactions in cultivating emotional solidarity. For example, positive interactions among cosplay tourists have been shown to promote emotional solidarity [19]. Furthermore, emotional solidarity has been found to mediate the relationship between the diversity and quality of host–guest interactions and tourists’ stereotypes [11]. Aleshinloye et al. (2020) demonstrated that the influence of emotional solidarity on place attachment and social distance varies with the frequency of interactions [20]. Recent investigations have also highlighted the role of individual traits and perceptions—such as emotional intelligence [21], perceived risk [22], and place attachment [23]—in shaping emotional solidarity. However, the ways in which the characteristics of specific destinations influence emotional solidarity remain unclear. This is particularly salient in rural tourism, where strong emotional connections are prevalent and the inherent characteristics of such destinations may catalyze emotional solidarity; however, empirical research in this area remains limited.

To investigate the effect of emotional solidarity in rural tourism, this study draws on the theory of halo effect and introduces two key dimensions: perceived authenticity and nostalgic emotion. On one hand, tourists actively seek nostalgic and authentic experiences in rural settings [24,25]. The nostalgia evoked by tourists in rural settings strengthens their emotional attachment to the destination [25]. Amidst the rapid urbanization in China, individuals value and yearn for emotional encounters in rural surroundings [6]. Consequently, according to the theory of halo effect, the authentic rural atmosphere and the nostalgic emotion it induces will become the psychological antecedents that transfer the tourists’ positive impression of the destination to the local residents, thus strengthening the emotional solidarity among them. On the other hand, drawing upon the cognitive–affect–behavior framework, a potential association between perceived authenticity and nostalgic emotions can be posited. Moreover, this positive relationship has been empirically validated in the context of heritage tourism [26]. Accordingly, this study seeks to extend these findings to rural tourism by examining the intention to revisit as the outcome variable, thereby highlighting its influence on tourist behavior.

In summary, this paper delineates the following research questions: (1) Does perceived authenticity positively influence nostalgic emotion in rural tourism? (2) Can perceived authenticity and nostalgic emotion serve as effective precursors to enhance emotional solidarity between tourists and residents in rural tourism? (3) Does rural tourists’ emotional solidarity with residents foster their revisit intention? To address these questions, we develop a pertinent theoretical framework and validate it by gathering quantitative survey data, leading to subsequent discussions grounded in the analysis outcomes. The findings also offer valuable insights into the antecedents and consequences of emotional solidarity among rural tourists and residents, bearing practical significance for destinations aiming to foster positive interactions between hosts and tourists and devise effective marketing strategies.

## 2. Literature review and hypotheses development

### 2.1. Cognition-affect-behavior (CAB) framework

The cognitive-affect-behavior (CAB) framework is effective in explaining the behavioral intentions of highly engaged consumers [27]. It demonstrates how consumers’ beliefs, perceptions, and thoughts elicit emotions and eventually influence behaviors [28]. By utilizing the CAB framework and the theory of halo effect, we developed a conceptual model to investigate the correlation between tourists’ perceived authenticity, nostalgic emotion, emotional solidarity, and revisit intentions.

**Fig 1:**
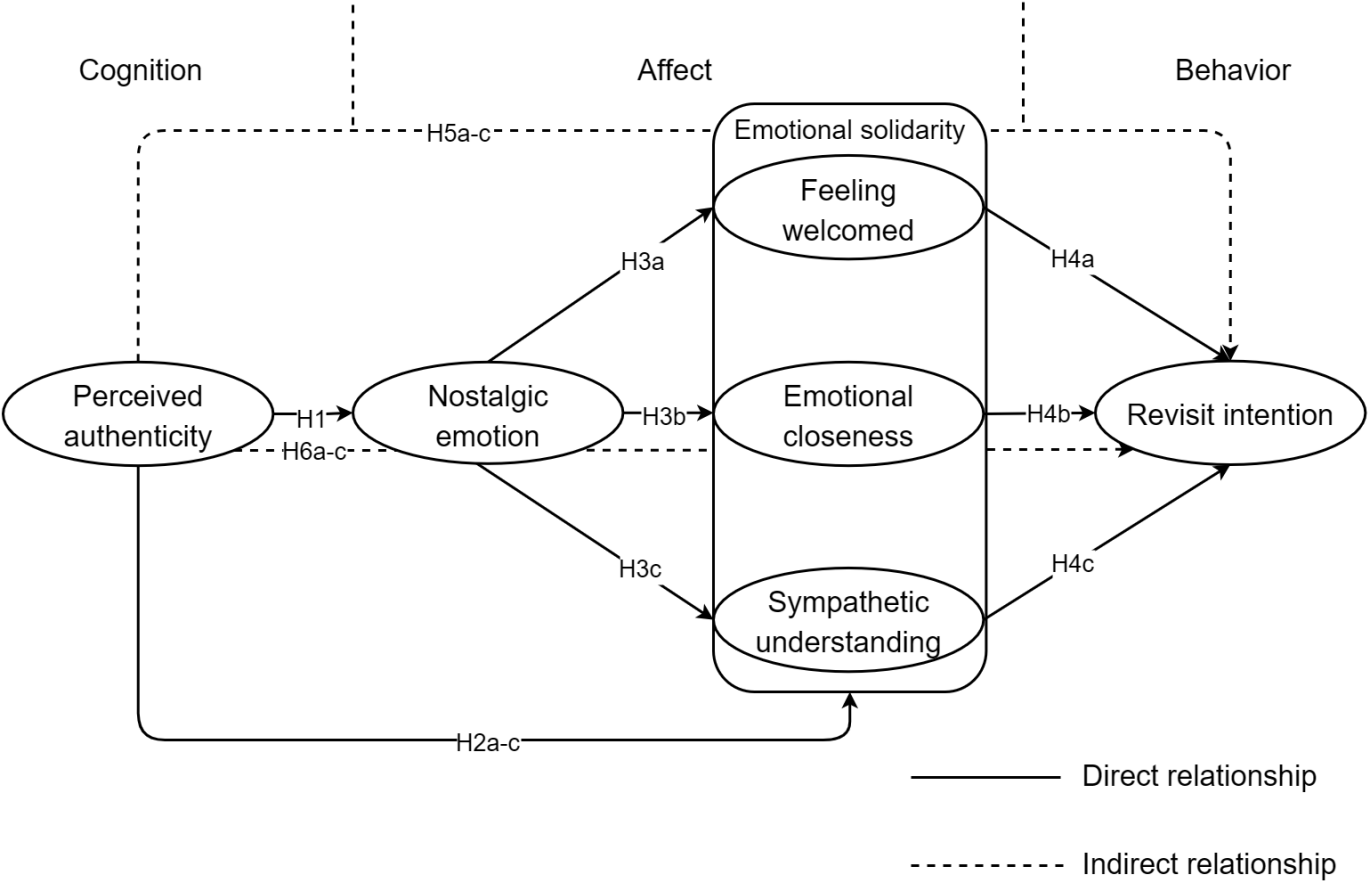
Schematic diagram of research hypotheses.

Perceived authenticity refers to an individual’s perceived authenticity formed based on his or her own expectations and thoughts, which can be influenced by environmental factors and varies according to the individual’s experience [29], thus constituting the cognitive component of the model. Nostalgia is an evoked emotional experience in the tourism context, and emotional solidarity represents the extent to which tourists and residents are emotionally connected [10,30], both of which together constitute the affective dimension of the model. Revisit intention, on the other hand, represents tourists’ intention to return to the destination as a behavioral response. The specific conceptual model is illustrated in Fig 1.

### 2.2. The theory of halo effect

The halo effect is a psychological phenomenon indicating that an individual’s judgement towards a particular target is swayed by external factors [31]. In other words, the evaluation of a single factor is influenced by other factors, precluding the isolated assessment of each factor [31,32]. Under the influence of the halo effect, individuals form judgements about others and objects based on known characteristics and conditions, where positive impressions engender a favorable halo, leading to a more positive evaluation of the target [33,34].

In tourism studies, a manifestation of the halo effect is observed where the image of a destination influences tourists’ evaluations of products and their purchasing decisions [35]. This effect is particularly pronounced among international tourists, as geographic distance makes the country of destination a halo for international tourists to evaluate its products, the image of a country directly or indirectly influences the evaluation of its products by tourists [36,37]. While the application of the theory of halo effect to the country-of-origin effect is well-established [38], as a widespread psychological phenomenon, it equally applies to the micro-environment of domestic tourism destinations [31]. Environmental cues surrounding tourists guide their perceptions of the destination’s image, thereby affecting their product evaluations [39], and residents, as integral to the destination, are inevitably perceived by tourists under the halo of the destination. Building upon Chartrand (2005)’s research, the process is summarized by Lee (2019) as such: when tourists encounter environmental cues, they form an image of the destination, which, in turn, affects their evaluation of things related to the destination [38,39].

In this study, we employ the theory of halo effect to elucidate the impact of rural tourists’ perceived authenticity of a destination on their emotional solidarity with residents. Specifically, when tourists perceive authentic rural charm, they will build up the traditional and retro rural image of the destination and generate nostalgic emotion. Such image and emotion will become a halo that makes them have a similar perception of local residents, that is, local residents have the positive characteristics of hospitality and willingness to interact with people in traditional Chinese agricultural society, so as to strengthen the emotional solidarity with the local residents.

### 2.3. Emotional solidarity

Durkheim (1995) originally conceptualized emotional solidarity as stemming from shared beliefs, communal behaviors, and interpersonal interactions, fostering a sense of solidarity among individuals [18]. The introduction of this concept into the tourism field by Woosnam and Norman (2010) underscored the importance of recognizing the potential for close relationships between tourists and residents [10]. Subsequently, they devised a scale to gauge emotional solidarity between residents and tourists, categorizing it into three dimensions: welcoming nature, emotional closeness, and sympathetic understanding [40]. Expanding on this groundwork, research by several scholars suggests that residents with stronger emotional connections to tourists exhibit more favorable attitudes and perceptions towards tourism development in their community [41].

This emotional bond can also be examined from the viewpoint of tourists [12]. Woosnam et al. (2018) studied the impact of place attachment in fostering emotional solidarity with residents [23]. Tan and Tsu (2024) discovered that the quality and variety of interactions with hosts can boost emotional solidarity and mitigate negative stereotypes associated with tourists [11]. Tang et al. (2023) highlighted the influence of peer-to-peer interactions within the cosplayer community on their emotional solidarity [19]. Emotional solidarity has the potential to forecast tourists’ behavioral intention, including consumption intention [13], loyalty [22], and intention to revisit [42]. Emotional solidarity among tourists is especially pronounced in forms of special interest tourism that involve visits to specific destinations or attractions, such as religious, heritage, cruise, and medical tourism [41,43]. In the context of rural tourism, visitors tend to place greater emphasis on interactions and emotional connections with hosts [6]. However, few studies have investigated the mechanisms underlying emotional solidarity in this setting. Additionally, the existing literature on the antecedents of emotional solidarity remains largely confined to the level of interpersonal interactions, with limited exploration of individual perceptions and emotional dimensions. Accordingly, this study investigated the facilitating effects of two key constructs in rural tourism—perceived authenticity and nostalgic emotions—on emotional solidarity between tourists and hosts.

### 2.4. Authenticity in rural tourism

Being one of the earliest and most debated concepts in tourism research, authenticity in tourism has been the focal point of extensive research and discourse [44]. Examined through various authenticity perspectives, many studies draw from the authenticity typologies introduced by Wang (1999), which categorize authenticity into objective authenticity, constructive authenticity, postmodern authenticity, and existential authenticity [45]. Constructive authenticity, also referred to as perceived authenticity [26], pertains to the authenticity individuals perceive based on their expectations and beliefs, shaped by environmental influences and exhibiting diverse features rooted in personal experiences [29,45]. This corresponds with the unique experiences of tourists influenced by various sources of information, rendering constructive authenticity the predominant concept in tourism authenticity research [44].

Authenticity has been investigated in various themes within the tourism sector, including cultural heritage, commodification, staging, marketing, tourist practices, and performance [44], with a specific focus on its importance in heritage tourism [46]. Moreover, authenticity plays a significant role in rural tourism [47]. The primary allure of rural areas is in offering visitors genuine rural experiences, where authenticity is viewed as an essential component of rurality [48]. Urry (2002) suggests that the tourist gaze prompts locals to reshape their identities according to tourists’ views on authenticity [49]. Consequently, in the course of tourism development, the commercialization of rural areas is unavoidable [50], underscoring the importance of conserving rural authenticity as a fundamental aspect of sustainable rural progress [51]. This conflict between commercialization and authenticity is especially prominent in China [52]. Owing to regional development discrepancies in China, rural areas showcasing objective authenticity are frequently aged and dilapidated, disregarded by tourists, whereas meticulously crafted picturesque rural locales are in high demand, exemplifying a staged rural idyll phenomenon, mirroring tourists’ quest for constructivist authenticity in rural tourism [53].

In this context, for tourists in the ‘front-stage,’ their views on rural authenticity are frequently subjective, shaped by their social and cultural milieu, along with personal encounters, thereby molding their tourism encounters [54,55]. Against this backdrop, this study explored the perceived authenticity of rural tourists and its influence on their emotions and behavioral intentions.

### 2.5. Nostalgia in rural tourism

Within the realm of tourism research, nostalgia is recognized as a driving factor for travel [30,56]. Individuals inclined towards nostalgia actively seek memorabilia linked to their cherished past, utilizing past emotions to provide solace [30]. Moreover, nostalgia has been conceptualized as an emotionally triggered encounter elicited through travel, aimed at examining its influence on behavior [30]. With the acceleration of urbanization, rural areas are also associated with nostalgia, evoking memories of a genuine and picturesque past [57]. The distinctive ambiance of rural areas could evoke nostalgic emotion in tourists, acting as a motivating factor for rural tourism [25]. Just like the study by Chen et al. (2014) on nostalgic emotion among restaurant customers, rural tourism may elicit sentiments of past experiences for tourists with rural upbringings, characterized as personal nostalgia or ‘real nostalgia [58].’ Young tourists without exposure to rural life may acquire knowledge about rural areas from the experiences of their predecessors and media, resulting in ‘simulated nostalgia’ or ‘collective nostalgia.’ For instance, the research suggests that individuals can experience intense nostalgic emotion towards destinations with which they have no direct affiliation [59]. Zhou’s (2014) study highlights that nostalgia, authenticity, and idealized rural settings collectively shape the rural destination image perceived by tourists [50]. However, few studies have explored the potential impact of rural authenticity on nostalgia and how nostalgia, for example, enhances the emotional connection between tourists and their hosts.

### 2.6. Hypotheses development

Nostalgia is a poignant longing for the past that is closely related to authenticity [60]. The significant impact of authenticity on nostalgic emotion has been effectively explored in heritage tourism [26,61], and the study also supports the influence of perceived destination authenticity on tourists’ nostalgic emotion [62]. Rural environments often possess inherent nostalgic characteristics [25,63], and tourism stakeholders focus on the authenticity of rural elements in order to evoke tourists’ nostalgic emotion [25]. Moreover, people seek authentic rural destinations because rural authenticity has the potential to trigger their nostalgic emotion [64]. Therefore, for tourists motivated by nostalgia in rural tourism, when their perception of the destination aligns more with their memories and past experiences, the emergence of nostalgic emotion is often inevitable [64]. Even if nostalgia is not the primary motivation for tourists, perceiving authentic rural characteristics may still evoke their imagination of the past. Based on this and the CAB framework, the following hypothesis is proposed:

**H1:** The perceived authenticity of rural tourists positively influences their nostalgic emotion.

Much evidence suggestes that authenticity can evoke tourists’ attachment to the destination, known as place attachment [65–67]. Empirical research found that place attachment is a significant predictor of emotional solidarity between tourists and residents [23]. Patwardhan et al. (2020) also revealed a direct impact of place attachment on emotional solidarity [22]. Some scholars have proposed that emotional solidarity may also be an antecedent of place attachment, suggesting a bidirectional relationship between the two [68]. All the above studies have shown the halo effect in the destination, that is, tourists’ emotions towards the destination or residents will influence each other, providing the possibility that tourists’ perceived authenticity precedes place attachment in forming emotional solidarity with residents in rural tourism settings. Furthermore, in rural tourism, tourists’ perceived authenticity (traditional lifestyle, architecture, etc.) may resonate with local residents, leading to shared beliefs and behaviors [68], such as village preservation and retention of village characteristics, and may serve as antecedents for fostering emotional solidarity [69]. Based on this and the CAB paradigm, the following hypothesis is proposed:

**H2:** The perceived authenticity of rural tourists positively influences their feeling welcomed (H2a), emotional closeness (H2b), and sympathetic understanding (H2c).

An inherent characteristic of nostalgia is its social nature [70], reflecting an emotional connection between individuals and places [25,71]. According to the theory of halo effect, when people are exposed to environmental cues, they will produce judgments and emotions, which will affect their evaluation of things related to the destination [39]. For tourists, nostalgic emotion caused by rural authenticity is related to their attitude toward local residents. Furthermore, psychological literature indicates that nostalgic emotion also plays a role in interpersonal connections, with recent research suggesting that nostalgic emotion can alleviate social anxiety and enhance social interaction skills [72]. Large-scale testing has established a positive impact of individual nostalgic tendencies on empathy levels and prosocial behavior [73]. Furthermore, recent studies have demonstrated that nostalgia is an effective means of strengthening intergroup relationships [74], as individuals experiencing nostalgia may perceive those around them as part of a broader collective. These studies provide reasons to believe in the positive effects of nostalgic emotion on the interpersonal relationships between rural tourists and residents. Therefore, the following hypothesis is proposed:

**H3:** The nostalgic emotion of rural tourists positively influences their feeling welcomed (H2a), emotional closeness (H2b), and sympathetic understanding (H2c).

A substantial amount of empirical research indicates that emotional solidarity often foreshadows positive behavioral intention. For example, emotional solidarity significantly predicts residents’ attitudes and behavioral intention towards supporting tourism development [75,76]. Establishing positive relationships with tourists also helps foster residents’ positive perceptions of the impact of festivals [23]. Similarly, for tourists, emotional solidarity with residents also has similarly positive effects. One of the most well-known impacts is the influence of emotional solidarity on tourist satisfaction and loyalty [22,77,78], as well as its influence on tourist value co-creation behavior [79], environmental responsibility behavior [80], and image perception of destination [41], among a range of other outcomes. Based on this and the CAB model, the following hypothesis is proposed:

**H4:** The feeling welcomed (H4a), emotional closeness (H4b), and sympathetic understanding (H4c) of rural tourists positively influence their revisit intention.

Finally, incorporating the above hypotheses, this study also proposes that nostalgic emotion and emotional solidarity mediate the relationship between rural tourists’ perceived authenticity and their revisit intention. All hypotheses are presented in Fig 1.

**H5:** The feeling welcomed (H5a), emotional closeness (H5b), and sympathetic understanding (H5c) of rural tourists mediate the relationship between their perceived authenticity and revisit intention.

**H6:** The nostalgic emotion and the feeling welcomed (H6a), emotional closeness (H6b), and sympathetic understanding (H6c) of rural tourists play a chain mediating role between their perceived authenticity and revisit intention.

## 3. Research design

### 3.1. Variables and measurement

The items used in this survey were drawn from previously validated scales, enhancing the robustness of the measurement. In designing the questionnaire, the measurement items under different variables were randomly arranged to reduce common method bias, thus enhancing the reliability of the measurements in this survey. The questionnaire is divided into three parts: 1) Introduction; 2) Measurement items; and 3) Demographic items.

Perceived authenticity refers to the scales of Gao et al. (2020) and Lu (2015) and consists of a total of 5 items [26,81]. Nostalgic emotion consists of a total of 8 items and refers to the study of Chen et al. (2014) [58]. Emotional solidarity consists of 3 subdimensions of feeling welcomed, emotional closeness, and sympathetic understanding, with a total of 12 items from the studies of Lai and Hitchcock (2017), Woosnam (2012), and Lai and Wong (2024) [82–84], and the 3 items of revisit intention were adapted from the study by Rather (2021) [85]. Therefore, the scale utilized in this study comprises a total of 6 primary dimensions and 28 items. All items are measured on a 5-point Likert scale (1 = strongly disagree; 5 = strongly agree).

As most items in the scale are from English literature, the questionnaire was primarily designed in English and then translated into Chinese by professional translators. Discussions were conducted with a professor specializing in tourism management and two graduate students to refine the wording of individual questionnaire items. This process involved correcting grammatical errors and improving sentence structure, which enhanced the clarity and appropriateness of the questionnaire statements.

### 3.2. Sample and data collection

Using Kock and Hadaya’s (2018) method to estimate the required sample size, it is recommended to have a minimum of 5 times the number of items [86]. With the current study having 28 items, at least 140 respondents are needed for the sample.

The questionnaire was distributed in January 2024 on the Credamo platform to Chinese domestic participants. Credamo is a professional questionnaire survey platform with over three million online samples. Compared to on-site surveys, online surveys can include visitors from more sources, ensuring the universality and representativeness of the samples. After being asked to recall their previous rural tourism experiences, participants filled out the questionnaire. This study ensured the anonymity of the questionnaire, informed all respondents of the study’s purpose, and obtained their written consent to participate in the survey. Additionally, we provided an explanation of the study’s purpose in the introduction section of the questionnaire, accompanied by the statement: By completing the questionnaire, you are agreeing to participate in the study. This research solely involved the questionnaire survey and did not include human clinical trials or animal experiments; therefore, the ethics committee of the corresponding author’s institution waived approval of this study. After excluding responses with insufficient answer time and those failing the attention check, 324 valid questionnaires were obtained.

Regarding the overall characteristics of the valid sample (Table 1), there were more female respondents than male respondents, with 186 females accounting for 57.4% and 138 males accounting for 42.6%. 74.4% of the respondents were aged between 18 and 45. The most common monthly income range was 5,000–8,000 RMB, accounting for 54.3%. In terms of educational attainment, 73.5% of respondents held a bachelor’s or associate’s degree, while 13.9% had a high school or vocational school diploma. Additionally, 43.5% of respondents were employed in enterprises, and 23.8% were students.

**Table 1:** The profiles of the respondents (N = 324).

Demographics	n	%
**Gender**		
Male	138	42.6
Female	186	57.4
**Age**		
18 ~ 24	99	30.6
25 ~ 40	194	59.9
41 ~ 60	30	9.3
61 and above	1	0.3
**Education**		
Less than high school	2	0.6
High school/ Technical school	45	13.9
Undergraduate/Associate degree	238	73.5
Postgraduate degree	39	12
**Occupation**		
Student	77	23.8
Civil servants	11	3.4
Government agencies and institutions	63	19.4
Enterprises	141	43.5
Freelance	15	4.6
Individual businesses operator	6	1.9
Others	11	3.4
**Average monthly income**		
Less than RMB 3,000	73	22.5
RMB 3,001–5,000	82	25.2
RMB 5,001–8,000	71	21.8
RMB 8,001–10,000	46	14.2
More than RMB 10,000	52	16

## 4. Results

### 4.1. Common method bias

Harman’s one-way test was conducted to check for common method bias prior to data analysis, and the results showed that the percentage of variance explained by the unrotated first common factor was 40.05%, which was below the threshold of 50%. Thus, no serious common method bias was present in this study [87]. In addition, the variance inflation factor (VIF) analysis revealed that the VIF values among variables were less than 1.388, remaining well below the threshold of 3.3, indicating that no serious multicollinearity issues were present in this study [88].

### 4.2. Measurement model

The model measurement utilized the PLS-SEM method in Smart-PLS 4.0. Compared to traditional CB-SEM, PLS-SEM is more suitable for exploratory research of theories and can handle more complex models. Additionally, PLS-SEM does not require data to adhere to a normal distribution [89]. The data analysis results (Table 2) demonstrate that the overall Cronbach’s α and the composite reliability values for all dimensions exceed 0.7, indicating a high level of reliability of the measurement model [90]. The standardized factor loadings of all measurement items exceed the threshold of 0.6 [91], and the average variance extracted (AVE) values for all variables exceed the threshold of 0.5, indicating a good level of convergent validity of the variables [92]. Finally, as shown in Table 3, the HTMT values for all variables are below the threshold of 0.85, confirming the model’s excellent discriminant validity [89].

**Table 2:** Measurement model results.

Dimension/Item	Loading	CR	AVE	Alpha
**Perceived authenticity**		0.889	0.692	0.889
In my recent rural travel experience, I thought the destination was well preserved.	0.833			
In my recent rural travel experience, I thought the destination presented local history and culture well.	0.833			
In my recent rural travel experience, I thought the destination was antiquated.	0.836			
In my recent rural travel experience, I thought the destination stayed its original features.	0.816			
In my recent rural travel experience, I thought the destination was authentic.	0.843			
**Nostalgic emotion**		0.934	0.682	0.933
In my recent rural travel experience, I can feel the serenity of simple life.	0.852			
In my recent rural travel experience, I can search for some feeling within me.	0.832			
In my recent rural travel experience, I can revisit my childhood.	0.837			
In my recent rural travel experience, I can remember how things used to be.	0.851			
In my recent rural travel experience, I can feel the memories in the life of past era.	0.823			
In my recent rural travel experience, I can feel the past is better.	0.798			
In my recent rural travel experience, I can feel the changing of time.	0.805			
In my recent rural travel experience, I can remember something from the past.	0.809			
**Feeling welcomed**		0.852	0.691	0.851
In my recent rural travel experience, I was proud to be welcomed as a visitor to the destination.	0.838			
In my recent rural travel experience, I felt residents appreciate the social benefits associated with my coming to the community.	0.815			
In my recent rural travel experience, I felt residents appreciated the contribution we (as visitors) make to the local economy.	0.822			
In my recent rural travel experience, I treated local residents fairly.	0.849			
**Emotional closeness**		0.850	0.687	0.848
In my recent rural travel experience, I felt like contacting some local residents.	0.827			
In my recent rural travel experience, I felt close to some local residents I have met.	0.828			
In my recent rural travel experience, I felt like making friends with some local residents.	0.810			
In my recent rural travel experience, I felt like interacting with some local residents.	0.850			
**Sympathetic understanding**		0.869	0.716	0.868
In my recent rural travel experience, I understood local residents.	0.831			
In my recent rural travel experience, I identified with local residents.	0.869			
In my recent rural travel experience, I felt affection toward local residents.	0.862			
In my recent rural travel experience, I had a lot in common with local residents.	0.823			
**Revisit intention**		0.830	0.745	0.829
I tend to visit the rural destination again.	0.846			
I think I will come back to the rural destination in the near future.	0.874			
I would love to come to the rural destination again.	0.870			

**Table 3:** Discriminant validity test results (HTMT criteria).

	RVI	FW	SU	NE	EC	PA
**RVI**						
**FW**	0.525					
**SU**	0.569	0.521				
**NE**	0.583	0.61	0.519			
**EC**	0.534	0.515	0.523	0.557		
**PA**	0.589	0.549	0.468	0.509	0.504	

PA: Perceived authenticity, NE: Nostalgic emotion, FW: Feeling welcomed, EC: Emotional closeness, SU: Sympathetic understanding, RVI: Revisit intention

### 4.3. Structural model

Based on VIF, *Q*^*2*^, and *R*^*2*^, the fit of the structural model can be evaluated (Table 4). The VIF values of all variables range from 1 to 1.388, all less than 3 [89], indicating a lack of significant multicollinearity issues in this study. The *Q*^*2*^ values for each dimension are exceed 0, suggesting good predictive relevance of the model [89]. The R^2^ values for all the endogenous variables are greater than 0.2. In consumer behavior studies, *R*^*2*^ values higher than 0.2 are considered satisfactory [89]. The *f*^2^of all variables are greater than 0.02, indicating that the effect size of each path is small or medium [93].

**Table 4:** Results of the structural model.

Hypotheses	Paths	β	*f* ^*2*^	*t*	Results
H1	PA - > NE	0.464	0.275	10.723	S
H2a	PA - > FW	0.288	0.102	4.952	S
H2b	PA - > EC	0.267	0.081	4.636	S
H2c	PA - > SU	0.248	0.066	4.229	S
H3a	NE - > FW	0.411	0.207	7.218	S
H3b	NE - > EC	0.374	0.158	6.716	S
H3c	NE - > SU	0.352	0.133	6.419	S
H4a	FW - > RVI	0.216	0.051	3.654	S
H4b	EC - > RVI	0.226	0.056	3.766	S
H4c	SU - > RVI	0.286	0.089	4.681	S

PA: Perceived authenticity, NE: Nostalgic emotion, FW: Feeling welcomed, EC: Emotional closeness, SU: Sympathetic understanding, RVI: Revisit intention

**Fig 2:**
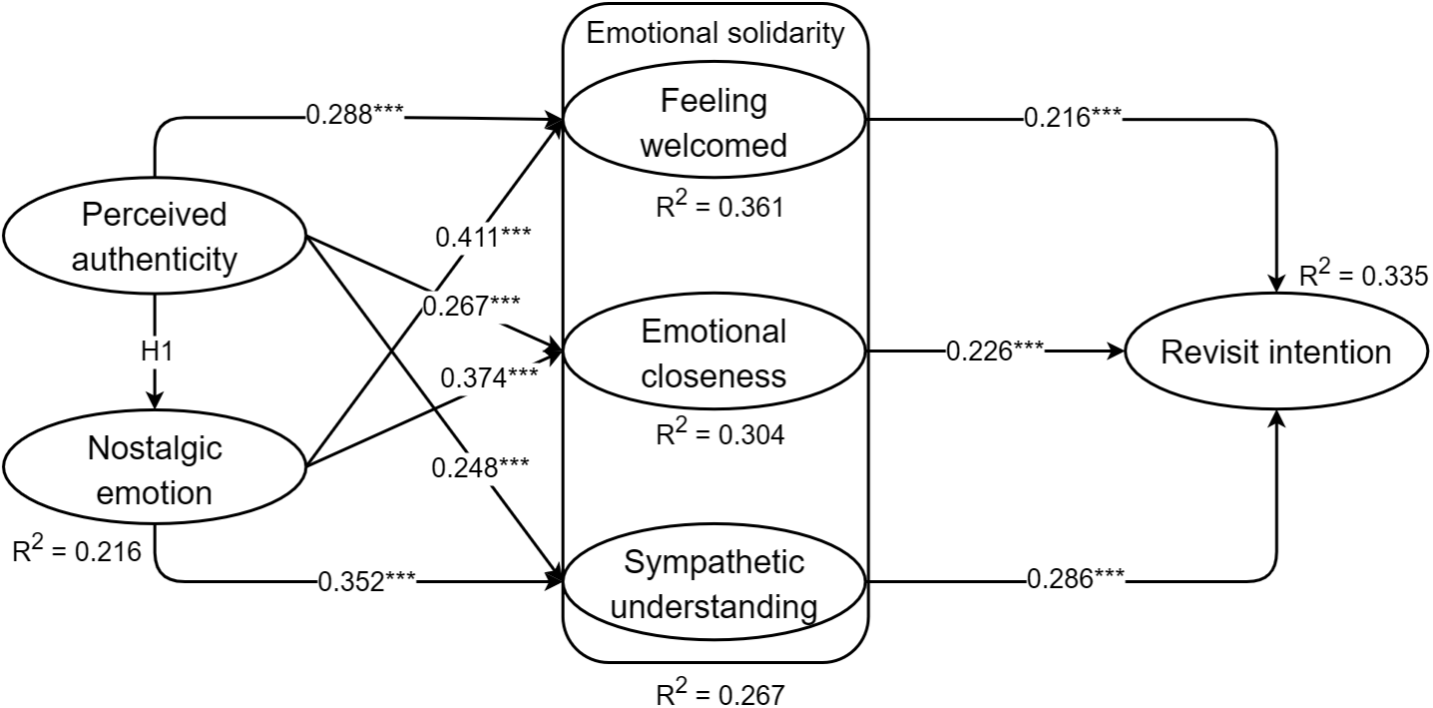
Structural model path coefficients.

Subsequent examinations were conducted to test the model assumptions, revealing that all hypotheses were supported (Fig 2). Finally, we employed bootstrap analyses, with the number of bootstrap samples set to 5000 and confidence level at 95%, to assess the mediating role of the nostalgic emotion and sub-dimensions of emotional solidarity between perceived authenticity and revisit intention (Table 5). The results indicate that feeling welcomed, emotional closeness, and sympathetic understanding all mediate the effect of perceived authenticity on revisit intention. Nostalgic emotion, on the other hand, jointly acts as a chain mediator with the sub-dimensions of emotional solidarity between perceived authenticity and revisit intention, confirming both H5 and H6.

**Table 5:** Results of the mediating test.

Hypotheses	Paths	β	*t*	Results
H5a	PA - > FW - > RVI	0.062	2.990	S
H5b	PA - > EC - > RVI	0.060	2.770	S
H5c	PA - > SU - > RVI	0.071	2.937	S
H6a	PA - > NE - > FW - > RVI	0.041	2.813	S
H6b	PA - > NE - > EC - > RVI	0.039	3.012	S
H6c	PA - > NE - > SU - > RVI	0.047	3.433	S

PA: Perceived authenticity, NE: Nostalgic emotion, FW: Feeling welcomed, EC: Emotional closeness, SU: Sympathetic understanding, RVI: Revisit intention

## 5. Conclusions and discussion

### 5.1. Discussion

First, the research findings indicate that rural tourists’ perceptions of authenticity can evoke nostalgic emotions. Gao et al. (2020) found that, in heritage tourism, tourists’ perceptions of authenticity exert a significant positive impact on nostalgia [26]. Moreover, distinctive features and cues present in rural environments may trigger nostalgic feelings among tourists [25]. Furthermore, nostalgia serves as an important motivator for rural tourists. Christou et al. (2018) also found that rural tourists’ nostalgic emotions are primarily elicited by a focus on “authenticity” and place-related elements [25]. Meng et al. (2021) identified a bidirectional relationship between nostalgia and authenticity in rural tourism [94]. Their study revealed that nostalgia is a key driving force for rural tourists, whereas the authenticity of rural areas facilitates the fulfillment of nostalgia, thus serving as an important attracting factor. Based on the aforementioned qualitative findings, the present study confirms the positive impact of authenticity on nostalgic emotions in rural tourism, thereby corroborating previous conclusions and providing additional empirical evidence.

In addition, both perceived authenticity and nostalgia significantly influence emotional solidarity. Previous studies have confirmed the predictive role of perceived authenticity on place attachment [65–67]. The present study extends this perspective by demonstrating that authenticity not only strengthens tourists’ emotional attachment to the destination but also fosters their emotional connection with rural residents. Moreover, relevant psychological literature suggests that nostalgic emotions strengthen interpersonal relationships and promote prosocial tendencies [72,73]. The present study validates this conclusion within the context of rural tourism, indicating that nostalgic emotions are an effective antecedent in promoting emotional solidarity between hosts and tourists.

Finally, all three dimensions of emotional solidarity—feeling welcomed, emotional closeness, and empathetic understanding—positively influence tourists’ intention to revisit, with empathetic understanding exerting the strongest effect. This finding supports the important predictive role of emotional solidarity on tourist satisfaction and loyalty, as identified in previous studies [95]. However, in contrast to the findings of Ribeiro et al. (2018), emotional closeness was found to have a significant positive effect on revisit intention in the present study [95]. This suggests that, within the Chinese cultural context, the motivations of rural tourists extend beyond sightseeing and nostalgia to include emotional exchange with hosts. Therefore, high-quality host–guest interactions emerge as a crucial factor in stimulating revisit intention.

### 5.2. Theoretical implications

First, this study explored the relationship between perceived authenticity and nostalgic emotion in the context of rural tourism, expanding the existing literature on authenticity. Previous research primarily focused on investigating how perceived authenticity influences nostalgic emotion in heritage tourism settings, with limited empirical studies examining this relationship in rural tourism. This study expanded the scope of perceived authenticity and confirmed its positive impact on tourists’ nostalgic emotion in rural tourism. This finding is in line with earlier research in heritage tourism [26,96,97], suggesting that an authentic rural environment can evoke personal or collective memories in tourists, eliciting their nostalgic emotion, which may vary among individuals and encompass personal, collective, and simulated nostalgia. While consistent with previous perspectives [25,64], empirical validation of this relationship remains limited.

Second, on the foundation of the CAB paradigm and the theory of halo effect, by linking perceived authenticity, nostalgic emotion, and emotional solidarity, this study not only demonstrated the plausibility of the three-dimensional relationship of host–tourist–place, but also effectively explored the antecedents of tourists’ emotional solidarity with residents in the context of rural tourism. This research goes further by including the psychological and emotional factors of the tourists, i.e., rural tourists’ perceived authenticity of place and nostalgic emotion are also positive predictors of their emotional solidarity, indicating the existence of a link between the host-tourist relationship and place [23]. This linkage is manifested in the fact that destinations not only stimulate perceived authenticity and promote emotional connections between tourists and places but also transfer this emotion to local residents, thereby promoting emotional solidarity, which in turn drives tourists to similar destinations. This conclusion is based on the discussion of the three-dimensional relationship of host–tourist–place on the basis of the one-dimensional relationship of human-place, indicating that this study has theoretical significance for enriching the three-dimensional relationship of host–tourist–place.

Furthermore, this study offers additional evidence for the halo effect at the micro level. Previous research has predominantly adopted a macro perspective, highlighting the halo effect of national image on the evaluation of tourism products [31,98]. Such studies are based on the assumption that a more favorable product image leads to higher purchase intentions and thus advocate leveraging national image to promote products [38]. This study, however, focuses on the destination itself and finds that the authenticity of rural areas influences tourists’ perceptions of local residents. This demonstrates that specific destination images can facilitate the formation of the halo effect. This conclusion enriches the existing literature on the halo effect in tourism contexts and provides a novel perspective on the mechanisms underlying the formation of host–guest relationships.

### 5.3. Managerial implications

Stakeholders in rural tourism should acknowledge that tourists’ nostalgia can be nurtured through the presentation of authenticity, fostering social interactions and the emotional connection between tourists and place [25]. Thus, in contrast to excessive tourism development and commercialization, presenting the genuine lifestyles of local residents and allocating resources for the preservation and enhancement of traditional structures to establish a nostalgic ambiance could be a more suitable strategy. Marketers can also further enrich the portrayal of the local historical charm on websites of tourist destinations by incorporating photos and promotional slogans to appeal to prospective nostalgic visitors.

Furthermore, the perception and emotion of rural tourists towards the destinations can also play a crucial role in cultivating emotional solidarity with the hosts. Studies demonstrate that tourists’ perceived authenticity of the destination and the nostalgic emotion it evokes can improve interaction and communication between tourists and local residents. Consequently, stakeholders should capitalize on this motivation by fostering tourists’ favorable perception of the destination to enhance their inclination for interaction. Organizing a variety of activities to bridge the gap between tourists and hosts is essential. Residents and managers who directly interact with tourists should be incorporated into the regulatory framework, prompting them to actively engage with and support tourists in portraying a welcoming host image, thereby making tourists feel appreciated.

Lastly, destination managers must grasp that nurturing emotional solidarity between tourists and hosts is a gradual process, not an immediate outcome. Interactions between tourists and hosts should be genuine and cordial, requiring training for residents and businesses to understand the significance of tourists in local progress, guiding them to sincerely care for and respect tourists, rather than focusing solely on profit-driven, one-sided transactions. It is crucial to avoid excessive promotion that could alienate tourists. While additional interaction after welcoming tourists is suitable, it is appropriate to be sensitive and recognize that establishing resonance and mutual understanding with every tourist may not always be achievable.

### 5.4. Limitations and future research

Undoubtedly, this study also possesses inherent limitations. First, although ethnic differences may exist between tourists and hosts in this research, they are all situated within the Chinese context. Therefore, the model utilized in the study could be further validated in destinations where tourists of diverse nationalities and ethnicities form the visitor base. Second, the study design is cross-sectional and utilizes non-probability sampling for sample collection. Subsequent studies could enhance the applicability of research findings by employing more robust mixed research methods and sampling techniques. Moreover, this study comprehensively explores the relationship mechanisms involving perceived authenticity, nostalgic emotion, and emotional solidarity, potentially neglecting the micro aspects of individual dimensions. However, the emotional changes experienced by tourists represent a dynamic process, and subsequent research could examine both the vertical fluctuations and horizontal variations in emotional solidarity between rural tourists and residents across diverse tourism contexts. For instance, it is valuable to investigate whether emotional solidarity between tourists and residents is compromised by the digitalization of rural destinations. Finally, nostalgia can be categorized into individual and collective forms, and the extent to which these distinct types of nostalgia influence the stimulation of emotional solidarity warrants further investigation.

## Supporting information

S1 Data Questionnaire. (DOCX)

S2 Data Questionnaire data. (XLSX)
