# Supplementary material for: Destination authenticity as a halo? Enhancing emotional solidarity with residents in rural tourism
Source: PLoS One. 2025 Sep 5;20(9):e0331644. doi: 10.1371/journal.pone.0331644 (PMC12412927; doi:10.1371/journal.pone.0331644)
Supplement: S1 Data — (DOCX) [file pone.0331644.s001.docx]

Dear Madam/Sir,

We are a research team from the School of Tourism at Northwest Normal University, conducting a study on the psychological and behavioral intentions of rural tourists concerning their perceived authenticity, nostalgic emotion, emotional solidarity with the residents, and revisit intention.

We would like to request your participation in filling out this questionnaire, which will only take a few minutes of your time. Please be assured that this questionnaire is anonymous, and all data collected will be used solely for academic research purposes. There are no right or wrong answers, please respond based on your genuine feelings.

**By answering and submitting this questionnaire, you will be deemed to know and agree to the above and participate voluntarily. If you do not wish to participate in this study, you may withdraw your participation.**

Thank you for your cooperation.

**Have you participated in rural tourism in six months?**

| □Yes □No |
| --- |

**Part A: Measurement items**

**Please mark "√" on the option you think is appropriate.**

(1 = strongly disagree; 2 = disagree; 3 = neutral; 4 = agree; 5 = strongly agree).

| **Perceived authenticity** | 1 | 2 | 3 | 4 | 5 |
| --- | --- | --- | --- | --- | --- |
| In my recent rural travel experience, I thought the destination was well preserved. | 1 | 2 | 3 | 4 | 5 |
| In my recent rural travel experience, I thought the destination presented local history and culture well. | 1 | 2 | 3 | 4 | 5 |
| In my recent rural travel experience, I thought the destination was antiquated. | 1 | 2 | 3 | 4 | 5 |
| In my recent rural travel experience, I thought the destination stayed its original features. | 1 | 2 | 3 | 4 | 5 |
| In my recent rural travel experience, I thought the destination was authentic. | 1 | 2 | 3 | 4 | 5 |
| **Nostalgic emotion** | 1 | 2 | 3 | 4 | 5 |
| In my recent rural travel experience, I can feel the serenity of simple life. | 1 | 2 | 3 | 4 | 5 |
| In my recent rural travel experience, I can search for some feeling within me. | 1 | 2 | 3 | 4 | 5 |
| In my recent rural travel experience, I can revisit my childhood. | 1 | 2 | 3 | 4 | 5 |
| In my recent rural travel experience, I can remember how things used to be. | 1 | 2 | 3 | 4 | 5 |
| In my recent rural travel experience, I can feel the memories in the life of past era. | 1 | 2 | 3 | 4 | 5 |
| In my recent rural travel experience, I can feel the past is better. | 1 | 2 | 3 | 4 | 5 |
| In my recent rural travel experience, I can feel the changing of time. | 1 | 2 | 3 | 4 | 5 |
| In my recent rural travel experience, I can remember something from the past. | 1 | 2 | 3 | 4 | 5 |
| **Feeling welcomed** |  |  |  |  |  |
| In my recent rural travel experience, I was proud to be welcomed as a visitor to the destination. | 1 | 2 | 3 | 4 | 5 |
| In my recent rural travel experience, I felt residents appreciate the social benefits associated with my coming to the community. | 1 | 2 | 3 | 4 | 5 |
| In my recent rural travel experience, I felt residents appreciated the contribution we (as visitors) make to the local economy. | 1 | 2 | 3 | 4 | 5 |
| In my recent rural travel experience, I treated local residents fairly. | 1 | 2 | 3 | 4 | 5 |
| **Emotional closeness** |  |  |  |  |  |
| In my recent rural travel experience, I felt like contacting some local residents. | 1 | 2 | 3 | 4 | 5 |
| In my recent rural travel experience, I felt close to some local residents I have met. | 1 | 2 | 3 | 4 | 5 |
| In my recent rural travel experience, I felt like making friends with some local residents. | 1 | 2 | 3 | 4 | 5 |
| In my recent rural travel experience, I felt like interacting with some local residents. | 1 | 2 | 3 | 4 | 5 |
| **Sympathetic understanding** |  |  |  |  |  |
| In my recent rural travel experience, I understood local residents. | 1 | 2 | 3 | 4 | 5 |
| In my recent rural travel experience, I identified with local residents. | 1 | 2 | 3 | 4 | 5 |
| In my recent rural travel experience, I felt affection toward local residents. | 1 | 2 | 3 | 4 | 5 |
| In my recent rural travel experience, I had a lot in common with local residents. | 1 | 2 | 3 | 4 | 5 |
| **Revisit intention** |  |  |  |  |  |
| I tend to visit the rural destination again. | 1 | 2 | 3 | 4 | 5 |
| I think I will come back to the rural destination in the near future. | 1 | 2 | 3 | 4 | 5 |
| I would love to come to the rural destination again. | 1 | 2 | 3 | 4 | 5 |

**Part B: Demographics**

**1. Gender**

| □Male □Female |
| --- |

**2. Age**

| □18~24 □25~40 □36-45 □61 and above |
| --- |

**3. Education**

| □Less than high school □High school / Technical school  □Undergraduate/Associate degree □Postgraduate and above |
| --- |

**4. Occupation**

| □Student □Civil servants □Government agencies and institutions  □Enterprises □Freelance □Individual businesses operator |
| --- |
| □Others |

**5. Average monthly income**

| □Less than RMB 3,000 □RMB 3,001–5,000 □RMB 5,001–8,000 □RMB 8,001–10,000 □More than RMB 10,000 |
| --- |

| ***Thank you very much for your answer!*** |
| --- |
